# Exercise and mitochondrial remodeling to prevent age-related neurodegeneration

**DOI:** 10.1152/japplphysiol.00611.2022

**Published:** 2022-12-15

**Authors:** Colleen L. O’Reilly, Benjamin F. Miller, Tommy L. Lewis

**Affiliations:** ^1^Aging and Metabolism Research Program, Oklahoma Medical Research Foundation, Oklahoma City, Oklahoma; ^2^Oklahoma City Veterans Association, Oklahoma City, Oklahoma

**Keywords:** brain, exercise, mitochondria, mitochondria remodeling, neurodegeneration

## Abstract

Healthy brain activity requires precise ion and energy management creating a strong reliance on mitochondrial function. Age-related neurodegeneration leads to a decline in mitochondrial function and increased oxidative stress, with associated declines in mitochondrial mass, respiration capacity, and respiration efficiency. The interdependent processes of mitochondrial protein turnover and mitochondrial dynamics, known together as mitochondrial remodeling, play essential roles in mitochondrial health and therefore brain function. This mini-review describes the role of mitochondria in neurodegeneration and brain health, current practices for assessing both aspects of mitochondrial remodeling, and how exercise mitigates the adverse effects of aging in the brain. Exercise training elicits functional adaptations to improve brain health, and current literature strongly suggests that mitochondrial remodeling plays a vital role in these positive adaptations. Despite substantial implications that the two aspects of mitochondrial remodeling are interdependent, very few investigations have simultaneously measured mitochondrial dynamics and protein synthesis. An improved understanding of the partnership between mitochondrial protein turnover and mitochondrial dynamics will provide a better understanding of their role in both brain health and disease, as well as how they induce protection following exercise.

## INTRODUCTION

Mitochondria are dynamic organelles known for their role in energy supply; however, they are also involved in many other critical cellular processes including calcium handling, apoptosis, and more. A decline in the performance of mitochondria is a hallmark of aging and is implicated in the progression of neurodegenerative diseases including Alzheimer’s disease (AD), amyotrophic lateral sclerosis (ALS), and Parkinson’s disease (PD) (1, 2). The limited and generally ineffective treatment options available for these diseases highlight a need to develop potential interventions to prevent or delay the onset of neurodegeneration. Although the molecular and cellular mechanisms remain unknown, physical activity attenuates cognitive decline and delays the onset of neurodegenerative diseases (3, 4). Aerobic exercise improves mitochondrial function, via mitochondrial remodeling, in peripheral tissues, such as skeletal muscle, liver, and heart, but the effects of aerobic exercise on mitochondria of the brain are less clear. Due to the highly adaptable and dynamic nature of mitochondria in the brain, establishing the impact of aerobic exercise on brain mitochondria presents unique challenges. This mini-review highlights our current understanding of mitochondrial remodeling for brain health. We will describe techniques employed to measure mitochondrial remodeling and highlight potential pitfalls of current practices. Finally, we will provide evidence that aerobic exercise has the potential to improve mitochondrial remodeling, and thus function, in the brain.

### Normal Mitochondrial Function Is Necessary for Brain Health

Maintaining homeostasis in the brain and modulating brain activity both require vast amounts of energy estimated at ∼240 kcal/kg/day (5). These energy demands lead to a strong dependence on mitochondrial energy production in the brain and vulnerability to loss of aerobic energy production (6). Age-related neurodegeneration is accompanied by a decline in mitochondrial function and increased oxidative stress, with associated declines in mitochondrial mass, respiration capacity, and respiration efficiency. Mitochondrial deficiencies have also been demonstrated in the substantia nigra with PD (7) and the cortex of AD and Huntington’s disease (8). These declines in function are associated with a failure to optimize the shape and distribution of mitochondria throughout the neuron in ALS (9), AD (10, 11), and PD (12).

In regard to energy production, the aging brain has decreased electron transport chain (ETC) activity at complexes I and IV (13). Several neurodegenerative diseases also have altered oxidative phosphorylation (OXPHOS). For example, there is disruption of complexes I and IV in PD and AD pathogenesis (14), whereas Huntington’s disease has disruption of complex III (15). The aging brain also has increased reactive oxygen species (ROS) production that is thought to damage mitochondrial proteins, activate the mitochondrial permeability pore, and cause mutagenesis of mitochondrial DNA (mtDNA) leading to senescence and neurodegeneration (16). The resultant oxidative damage is proposed as a mechanism of neurodegeneration (17), with oxidative modifications to DNA, lipids, and proteins as part of the pathological processes in PD and AD (18). Mitophagy decreases with age in neurons (19) and is impaired in both AD (20) and PD (21), resulting in accumulation of damaged or dysfunctional mitochondria in the brain (20). Finally, impairments in markers of mitochondrial biogenesis have been demonstrated with both AD (22) and PD (23), whereas alterations in markers of mitochondrial dynamics have been found in models of several neurodegenerative diseases (24–26) with concurrent decreases in mitochondrial function.

The heterogeneity of cells in the brain makes this organ particularly difficult to study as energy demands, and how the demand is met, likely varies across and within cell types (27). Recent transcriptomic and proteomic analysis of different brain cell types has revealed unique metabolic phenotypes (28). Traditionally, neurons were thought to primarily rely on OXPHOS for energy production and possess low glycolytic capacity (29, 30). However, more recent work argues that neurons switch to, and rely on, neuronal glycolysis during times of high activity (31). In addition, neurons require the calcium-buffering capacity of mitochondria to regulate activity and energy production (32). As neurons are comprised of different structures and substructures, proper mitochondrial distribution is imperative for both energy supply and calcium homeostasis (33) furthering the complex nature of these cell types. Astrocytes, a type of glial cell, maintain lower OXPHOS activity but have higher glycolytic rates (34, 35). This high glycolytic capacity of astrocytes produces lactate that can then be used as metabolic fuel by neurons via the astrocyte-neuron lactate shuttle (31, 36). Microglia, brain-specific immune cells, contain all the machinery for both glycolysis and OXPHOS (37), but their bioenergetic preferences are activation-state dependent with resting microglia depending on OXPHOS and activated microglia relying on glycolysis (38). The different metabolic phenotypes of the various brain cells highlight a need to first understand how mitochondria function across the various cell types in the brain, to be able to determine their response to disease or intervention.

### Protein Turnover and Methods for Measurement

Protein turnover is the dynamic interaction of protein synthesis and protein breakdown that determines protein concentration. Mitochondria are not made de novo, but rather remodel and expand by the removal (breakdown) and addition (synthesis) of proteins. The making of mitochondrial proteins has been reviewed at length, and requires the coordination of nuclear and mitochondrial genomes, through peroxisome proliferator-activated receptor-γ coactivator-1α (PGC-1α). Mitochondrial protein breakdown is dependent primarily on one of two processes. The first involves mitochondrial proteases (e.g., Lon proteases) or the ubiquitin-proteasome system for the breakdown of damaged or misfolded proteins. The second process is the more widescale breakdown through mitophagy, which is strongly tied to the dynamic processes discussed in the *Mitochondrial Remodeling in the Brain* section below.

We have previously noted the shortcomings of traditional approaches to measuring mitochondrial protein synthesis (39). In summary, markers such as the activation of PGC-1α and mRNA expression do not capture the posttranscriptional regulation of mitochondrial protein biosynthesis. This consideration is important because stimuli that remodel mitochondria are almost always bioenergetic in nature. Therefore, these stimuli impact energetic or redox signaling, which regulates the energetically costly process of translation through proteins such as mammalian target of rapamycin, mTOR. Furthermore, mitochondrial protein concentrations only capture if net protein content increases or decreases, which does not capture remodeling in the absence of net changes. The same limitations apply to markers of mitochondrial protein breakdown. Static snapshots of markers, such as relative protein concentration by Western blot, have inherent limitations when trying to capture dynamic events where rates (change over time) are important.

An alternative to the measurement of static markers is the use of isotopic tracing for measuring protein turnover of mitochondria in the brain. The concept of isotope labeling is relatively simple in that there is a precursor pool of amino acids enriched with isotopes that incorporate into proteins so that the proteins become enriched as a product of time (giving a rate) (40). The isotope comes in the form of a labeled amino acid or heavy water (i.e., deuterium oxide). Because of the advancements in mass spectroscopy in the past 10–20 years, stable isotopes are primarily used over radioactive isotopes. The use of stable isotopes allows for bulk determination of protein synthesis, including mitochondrial proteins after subcellular fractionation (41) or the synthesis of individual proteins (42). Direct measures of protein breakdown are more challenging. In a relatively steady state, protein breakdown (and half-life) can be directly determined from protein synthesis since they are equal. However, during nonsteady states such as periods of mitochondrial remodeling because of an intervention or functional decline, we cannot assume that mitochondrial protein synthesis and breakdown are equal. During these periods, breakdown then must be estimated from synthesis rates and changes in mitochondrial protein concentrations over time (43). Although isotopes facilitate the direct measure of mitochondrial protein synthesis and breakdown, there are still challenges with measuring these processes in the brain because of the region-specific and cell-specific changes in mitochondrial remodeling. By combining isotopic tracing with Mito-Tag mice (44, 45) or the ability to introduce small tags such as the Halo-tag (46), future work may be able to determine synthesis and breakdown rates for individual mitochondrial proteins in specific cell types of the brain.

### Mitochondrial Dynamics and Methods for Measurement

Three mechanisms contribute to mitochondrial dynamics: trafficking, fusion, and fission. Trafficking is the process of moving a mitochondrion from one region of the cell to another. The transport of mitochondria occurs mainly along the microtubule network in a kinesin and dynein-dependent manner, but can also occur along other cytoskeletal tracks (47). Mitochondrial rho (Miro) (48), an outer mitochondrial membrane protein, and Milton (TRAK in mammals) (49), a mitochondrial-associated protein, are responsible for the specific regulation of mitochondrial transport by controlling the interaction of the mitochondrion with the shared transport machinery of the cell. Fusion is the process of two mitochondria becoming one larger mitochondrion and relies on three mitochondrial GTPase’s: inner membrane-localized optic atrophy-1, OPA1 (50), and outer membrane-localized mitofusions, MFN1 and MFN2 (51). Protein-protein interactions and posttranslational modifications can impact the proclivity for fusion, but OPA1 and MFN1/2 are thought to be the final mediators of inner and outer membrane fusion respectively. Fission, the process of breaking one larger mitochondrion into multiple smaller mitochondria, is surprisingly controlled by several proteins not specific to mitochondria. Dynamin-related protein-1, DRP1, is the major GTPase involved in mitochondrial fission but is mostly localized in the cytoplasm and must be recruited to the outer mitochondrial membrane for scission (52). This recruitment is mainly performed by four proteins: mitochondrial fission factor, MFF, fission-1, FIS1, mitochondrial elongation factor 1, MIEF1, and mitochondrial elongation factor 2, MIEF2 (53). MFF and FIS1 are the most abundant of these outer membrane-localized DRP1 receptors but are also found on peroxisomes (54). MIEF1 and 2 are thought to be specific to mitochondria but are relatively sparse in the cell (55). As with fusion, the activity of fission proteins can be modified to alter the propensity and/or rate of fission founding a complex system by which the mitochondrion’s structure and ultimately function can be regulated.

Several methods have been developed to assess the state of mitochondrial dynamics. To simplify, we will discuss two distinct categories of methods: single time-point (or static) versus multi-time-point (or dynamic). Single time-point methods operate under the assumption that measurements of gene or protein expression level at a particular point in time provide a good correlate for activity, and include techniques such as RT-PCR, Western blot, or immunocytochemistry (56, 57). The strengths of these techniques lie in their ease of use, simple sample preparation, and relatively low cost; however, their major weaknesses are the inability to observe and quantify changes that occur over time, and a lack of cell specificity. A slightly improved version of the single time-point method is the visualization of mitochondrial morphology with either fluorescent or electron microscopy (58, 59). These techniques allow for the quantification of the mitochondrion’s shape at a particular point in time and provide more confidence that the balance of fission or fusion has been pushed in one direction or the other. To overcome the lack of temporal resolution, live imaging of fluorescently labeled mitochondria has been implemented both in cultured cells and in vivo (60). This multi-time-point technique allows the experimenter to visualize mitochondrial dynamics over time in living cells, and calculate the rate or flux of mitochondrial dynamics in a particular cell. A more advanced version of this assay employs the use of a photoactivatable or photoconvertible fluorescent marker allowing for pulse-chase experiments and better resolution of fusion events (61, 62). Challenges of these multi-time-point techniques include difficult sample preparation, expensive equipment, and lower throughput. However, whenever possible multi-time-point experiments are preferable as they offer a more comprehensive understanding of the dynamic processes occurring in the cell.

### Mitochondrial Remodeling in the Brain

The methods discussed earlier have led to a few overarching viewpoints of mitochondrial remodeling in the brain. First, mitochondrial remodeling in the brain appears to be on average slower than in most other organs of the body. For both the entire proteome and the mitochondrial proteome, the average turnover is between two to five times slower than in organs such as the liver and kidney (42, 63, 64). Second, early work suggested that all mitochondrial proteins turnover together as entire mitochondria are removed by mitophagy (42, 63), but synthesis and degradation of individual mitochondrial proteins also occur within a mitochondrion (64, 65). Live imaging experiments have confirmed the process of local mitochondrial mitophagy in neurons (66), whereas protein tracer experiments show distinct rates of turnover for different mitochondrial proteins in the brain (42). The physiological context that drives the choice between these processes remains unclear and is a fascinating area for future investigation considering the recent work describing the presence of long-lived mitochondrial proteins. Many brain mitochondrial complexes are maintained in a functional state for months to years (65, 67, 68), and this suggests a strong interplay between mitochondrial biogenesis, protein turnover, and fusion.

Third, different cell types in the brain show distinct patterns and rates of mitochondrial remodeling. In general, mitotic and more transient cell types are found to have higher rates of mitochondrial protein turnover than postmitotic and longer-lived cells (69). However, these studies were performed in vitro, and may be influenced by the turnover of the cells themselves. Although no direct comparison of the rates of mitochondrial dynamics have been performed in different cell types of the brain, cell morphology plays an important role in how the cell regulates these processes. For instance, mitochondrial trafficking is more processive in neurons than in glia as mitochondria need to be transported over the larger distances found in neuronal processes (70). In regards to fission and fusion, mitochondria tend to form more reticular networks in glial cells (71) but in neurons, the different compartments (axons and dendrites) of the cell have more discrete mitochondria (72, 73).

Finally, the developmental stage of the cell also plays an important role in mitochondrial remodeling in the brain. Recent work on both mitochondrial protein turnover and mitochondrial dynamics argues that on average developing cells have higher rates than mature cells, whereas on average mature cells have higher rates than aging cells (41). This finding suggests a general slowing of these processes over the lifetime of the cell. However, there are some important considerations that have not yet been fully explored. For example, there is great heterogeneity in protein types so that although the average of all proteins might be slower with aging, there can be an equal number of proteins that increase as decrease turnover. Second, there is the consideration for the role that cell proliferation plays in determining protein turnover since the turnover of cells also requires turnover of proteins. Third, most methods do not account for the fraction of proteins that become resistant to turnover (i.e., aggregated) in their modeling. Fourth, these studies often switch back and forth between in vitro and in vivo models. To our knowledge, every report about the rates of mitochondrial protein turnover and mitochondrial dynamics using in vivo methods return substantially lower rates than those found in cell culture models. For instance, brain protein synthesis in vivo returned average half-lives of around 9 days, whereas mixed cultures of cortex had average half-lives of around 5 days (42, 69). On the mitochondrial dynamics side, mitochondrial trafficking is lower in vivo than in both cultured neurons and organotypic slices of brain, whereas fission and fusion rates have yet to be directly measured in the mammalian brain in vivo (74, 75). The simultaneous measurement of both mitochondrial protein turnover and mitochondrial dynamics within the same brain/cell by dynamic methods is a rich area for future investigation with important implications for many areas of biology ranging from developmental diseases to aging.

### Mitochondrial Remodeling and Neurodegeneration

There are several indications that mitochondrial remodeling is disrupted in neurodegenerative diseases. An imbalance of mitochondrial dynamics toward fission, as measured by protein marker levels, and mitochondrial density have been observed in both AD and PD and is reported to exacerbate the pathology (76). In several neurodegenerative diseases there is an upregulation of mitochondrial fission proteins and mitochondrial fragmentation that is believed to be an attempt to segregate damaged mitochondria through mitophagy (77). Reduction of mitochondrial biogenesis, measured through the number of mitochondria, is also associated with the pathogenesis of neurodegenerative diseases (78).

Alterations in brain mitochondrial dynamics, such as the knockout of MFN2 in the cerebellum (79) or silencing of OPA1 as modeled in cell lines (80), accelerate neurodegeneration through changes to mitochondrial structure and function (80). Recent work in cortical and hippocampal tissues of a mouse model of AD demonstrates that mitochondrial fission is activated via increased calcium/calmodulin dependent protein kinase kinase 2-AMP-activated protein kinase (CAMKK2-AMPK) pathway signaling. Activation of the AMPK-MFF-Unc-51 like autophagy activating kinase (ULK) pathway increases mitophagy reducing mitochondrial density as visualized by live imaging of fluorescently labeled mitochondria, microtubule-associated protein 1A/1B-light chain 3, LC3, and Lysosome-associated membrane glycoprotein 1, LAMP1. Mitochondrial loss is shown to be the primary event responsible for the loss of dendritic spines suggesting a link to the cognitive impairments found in the AD brain (81). Increased mitochondrial fission in AD is further supported by results from cultured human M17 neuroblastoma cells with amyloid-beta precursor protein (APP) overexpression (10), and primary neurons from the cortex and hippocampus of mice with AD (82) where gene expression of common markers of fission are upregulated and markers of fusion downregulated. Taken together, these findings strongly suggest that mitochondrial remodeling is involved in the pathogenesis of neurodegenerative diseases, and implementing interventions that alter mitochondrial remodeling, like aerobic exercise, should be a priority.

### Aerobic Exercise and Brain Health

The health effects of regular exercise on cardiovascular fitness and skeletal muscle function are well documented and include disease prevention and reduced all-cause mortality. The general impact of aerobic exercise on aging-related health deficits is reviewed elsewhere (83). Physical exercise is now recognized as a nonpharmacological strategy to prevent and counteract age-related brain decline and neurodegeneration (84). Aerobic fitness is associated with reduced brain tissue loss (85), stimulation of new brain cell growth (86), reduced cognitive decline in older adults (87), and is considered a protective mechanism against the development and pathogenesis of neurodegenerative diseases (88). Physical exercise also improves cognitive function (89) and many of the benefits of exercise to brain function are related to alterations of brain structure (90). In rodent models, exercise enhances synapse formation (91), long-term potentiation (92), and stimulates neurogenesis (92, 93). During aging, moderate regular aerobic exercise delays mitochondrial decline in the brain by counteracting the increased oxidative stress and decreased mitochondrial enzymatic activities (94). Aerobic exercise also enhances electron transfer system enzyme activity (94) and antioxidant capacities (94) in the brain. Interestingly, the impact of exercise on mitochondrial alterations mediated by aerobic exercise may be intensity-dependent (95). Age-related declines in mitochondrial function are an early and significant part of several neurodegenerative diseases. Exercise could mitigate many of these negative alterations to mitochondrial function (96) and delay progression of neurodegenerative diseases (4, 84). Epidemiological data suggest that those who maintain moderate exercise throughout their life are at reduced risk for AD (97, 98) and that there might be a dose-dependent impact of exercise on cerebral accumulation of Aß (99). Running wheel exercise attenuates the development of Aß pathology of the hippocampus and preserves cognitive function in mouse models of AD (100). In addition, 20 wk of treadmill training improved cognitive function, increased levels of complexes I, IV, and V activities, and decreased mitochondrial DNA damage in isolated mitochondria of the hippocampus (101). In a mouse model of PD, lesions of substantia nigra pars compacta were reduced when animals completed a chronic treadmill exercise program, and the exercising animals had a rescue of ATP content and respiratory control ratio (102). It has also been demonstrated that resistance exercise training reduces neuroinflammation (103, 104), attenuates neuropathological changes in a mouse model of AD (104), and improves mitochondrial health in the hippocampus (103). In addition, a mixed aerobic-strength training model induced miRNA changes in the brain of the 3×TG mouse model of AD (105). However, the literature investigating resistance exercise is currently limited, which highlights a need for more research in this area. Taken together, current findings strongly argue that exercise elicits functional adaptations in brain mitochondria to improve brain function.

### Aerobic Exercise and Mitochondrial Remodeling in the Brain

The impact of aerobic exercise on brain mitochondrial remodeling is much less defined than in other tissues such as skeletal muscle. In skeletal muscle, the consensus is that maintenance of mitochondrial homeostasis, both energetically and otherwise, requires coordinated regulation of mitochondrial dynamics and the quantity and quality of proteins. Eight weeks of aerobic exercise training in mice increased PGC-1α mRNA expression (106, 107) and protein (108). The increase in PGC-1α mRNA expression data was also noted after an acute bout of aerobic exercise (109). As discussed, there are clear limitations to concluding that these changes in PGC-1α resulted in changes to the turnover of mitochondrial proteins. As far as we are aware, no studies have made direct measures of mitochondrial protein turnover (e.g., with isotopic tracers) in rodents during exercise in health, age, or with neurodegeneration.

Investigation of exercise effects on mitochondrial dynamics of the brain are also lacking, and what is known relies heavily on gene expression and protein markers of mitochondrial dynamics at single time points. Treadmill training in a PD model reduced neuron loss and behavioral changes of the substantia nigra and striatum by increasing respiration, which was associated with increased protein expression of markers of mitochondrial dynamics (110). In addition, it has been demonstrated that chronic exercise training improved MFN1 and MFN2 content as well as reduced FIS1 content in rat hippocampus (111). Protein levels of OPA1, MFN2, and DRP1 were reduced in the substantia nigra and striatum of a mouse model of PD but were rescued by treadmill exercise (110). Finally, 12 wk of treadmill training increased the content of fusion proteins (OPA1 and MNF2) in the hippocampus of an Alzheimer’s disease mouse model (112). Although these findings highlight the fact that mitochondrial dynamics are an important part of neurodegenerative pathogenesis, they almost exclusively rely on static markers of dynamic processes and little has been done to confirm these findings with dynamic imaging.

Finally, although the two remodeling processes of mitochondrial protein turnover and mitochondrial dynamics and are often studied independently, they are typically coupled physiologically. For example, loss of Drp1 results in reduced mitophagy as mitochondria are too large to be engulfed (113). In addition, in AD, compromised mitophagy and the impaired ability to remove defective mitochondria is a pivotal event in progression of the disease and restoration of mitophagy ameliorates the pathogenesis (114). Studies of mitophagy in PD showed increased PTEN induced kinase 1 (PINK1) and sequestosome-1 (P62) (110) in the striatum that were normalized with treadmill exercise. Thus, treadmill training enhanced fusion and repaired mitophagy in PD, which together assisted in counteracting neurodegeneration. This finding strongly argues that both primary components of mitochondrial remodeling should be studied together for a complete understanding of how exercise improves brain health.

## CONCLUSIONS

**Figure 1.**
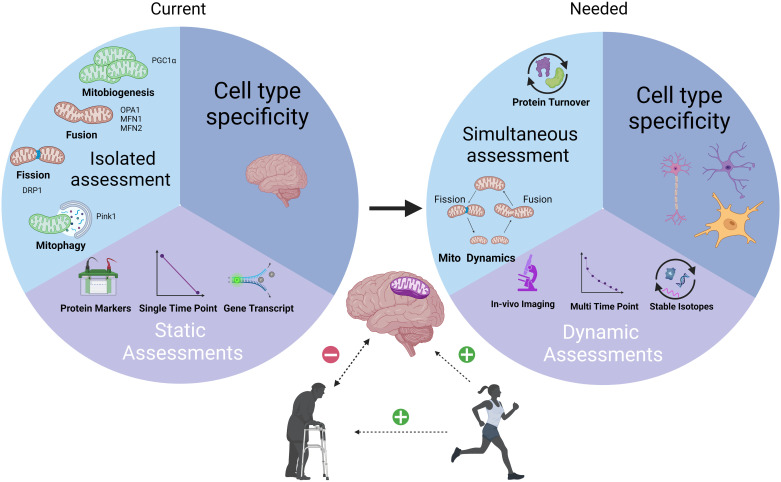
Schematic representation of current practices for analysis of mitochondrial remodeling. Aerobic exercise has positive effects on the healthy brain and may help slow or prevent neurodegeneration. A likely site of the positive benefit of aerobic exercise is the mitochondria. Aerobic exercise is known to cause mitochondrial remodeling. However, a current limitation to our understanding of mitochondrial remodeling in the brain is the reliance on static assessments that use markers for both mitochondrial dynamics and protein turnover that also lack cell type specificity. We advocate for additional studies that use methods that assess the dynamic nature of mitochondrial remodeling events. We also stress that studies should focus on the simultaneous assessment of both mitochondrial dynamics and protein turnover. Finally, we need to understand cell type specificity of these responses. Focusing effort on our proposed methodological approaches could accelerate our understanding of the relatively unknown mechanisms through which aerobic exercise positively impacts brain health. This figure was created with Biorender.

Aerobic exercise training elicits functional adaptations to improve brain health, and current findings suggest that mitochondrial remodeling plays an important role in these positive adaptations (Fig. 1). It will be important to assess different modalities of exercise, like resistance exercise, to provide a more comprehensive understanding of how exercise training can impact the brain. However, we note that caution is warranted when interpreting the data because we have very few direct measures of these dynamic processes. Therefore, the rate at which mitochondrial remodeling occurs, and how much that is accelerated with exercise training, remains largely unknown. We also identify a need for studies that couple measurements of both the rates of mitochondrial protein turnover and fission/fusion simultaneously since these processes are intricately linked. Finally, more work is required to understand the differences between the cell types present in the brain with respect to mitochondrial remodeling. Incorporation of in vivo, dynamic, multi-time-point experiments would enhance our understanding of the partnership between mitochondrial protein turnover and mitochondrial dynamics that improve brain health. This insight will allow for a better understanding of the dynamic pathogenesis of neurodegeneration, as well as the protective effects that exercise training provides to the brain.

## GRANTS

Dr. Miller’s work is funded by National Institute on Aging Grants R01AG074502, R01AG064951, and R01AG074551, and U.S. Department of Veterans Affairs Grant VA I01 BX005592. Dr. Lewis’s work is funded by National Institute of General Medical Sciences Grant R35GM137921. Drs. Miller and Lewis also received a seed grant from the Presbyterian Health Foundation that led to the current work.

## DISCLOSURES

B.F.M. is an editor of *Journal of Applied Physiology* and was not involved and did not have access to information regarding the peer-review process or final disposition of this article. An alternate editor oversaw the peer-review and decision-making process for this article. None of the other authors has any conflicts of interest, financial or otherwise, to disclose.

## AUTHOR CONTRIBUTIONS

C.L.O., B.F.M., and T.L.L.Jr. drafted manuscript; C.L.O., B.F.M., and T.L.L.Jr. edited and revised manuscript; C.L.O., B.F.M., and T.L.L.Jr. approved final version of manuscript.

## References

[1] Trigo D, Avelar C, Fernandes M, Sá J, da Cruz e Silva O. Mitochondria, energy, and metabolism in neuronal health and disease. FEBS Lett 596: 1095–1110, 2022. doi:10.1002/1873-3468.14298. 35088449

[2] McFarland R, Taylor RW, Turnbull DM. A neurological perspective on mitochondrial disease. Lancet Neurol 9: 829–840, 2010. doi:10.1016/S1474-4422(10)70116-2. 20650404

[3] Kou X, Chen D, Chen N. Physical activity alleviates cognitive dysfunction of Alzheimer’s disease through regulating the mTOR signaling pathway. Int J Mol Sci 20: 1591, 2019. doi:10.3390/ijms20071591. 30934958 PMC6479697

[4] Law C-K, Lam FM, Chung RC, Pang MY. Physical exercise attenuates cognitive decline and reduces behavioural problems in people with mild cognitive impairment and dementia: a systematic review. J Physiother 66: 9–18, 2020. doi:10.1016/j.jphys.2019.11.014. 31843427

[5] Wang Z, Ying Z, Bosy-Westphal A, Zhang J, Schautz B, Later W, Heymsfield SB, Müller MJ. Specific metabolic rates of major organs and tissues across adulthood: evaluation by mechanistic model of resting energy expenditure. Am J Clin Nutr 92: 1369–1377, 2010. doi:10.3945/ajcn.2010.29885. 20962155 PMC2980962

[6] Delettre C, Lenaers G, Griffoin J-M, Gigarel N, Lorenzo C, Belenguer P, Pelloquin L, Grosgeorge J, Turc-Carel C, Perret E, Astarie-Dequeker C, Lasquellec L, Arnaud B, Ducommun B, Kaplan J, Hamel CP. Nuclear gene OPA1, encoding a mitochondrial dynamin-related protein, is mutated in dominant optic atrophy. Nat Genet 26: 207–210, 2000. doi:10.1038/79936. 11017079

[7] Schapira AHV. Mitochondrial dysfunction in Parkinson’s disease. Cell Death Differ 14: 1261–1266, 2007. doi:10.1038/sj.cdd.4402160. 17464321

[8] Picard M, McEwen BS. Mitochondria impact brain function and cognition. Proc Natl Acad Sci USA 111: 7–8, 2014. doi:10.1073/pnas.1321881111. 24367081 PMC3890847

[9] Smith EF, Shaw PJ, De Vos KJ. The role of mitochondria in amyotrophic lateral sclerosis. Neurosci Lett 710: 132933, 2019. doi:10.1016/j.neulet.2017.06.052. 28669745

[10] Wang X, Su B, Siedlak SL, Moreira PI, Fujioka H, Wang Y, Casadesus G, Zhu X. Amyloid-beta overproduction causes abnormal mitochondrial dynamics via differential modulation of mitochondrial fission/fusion proteins. Proc Natl Acad Sci USA 105: 19318–19323, 2008. doi:10.1073/pnas.0804871105. 19050078 PMC2614759

[11] Selfridge JE, E L, Lu J, Swerdlow RH. Role of mitochondrial homeostasis and dynamics in Alzheimer’s disease. Neurobiol Dis 51: 3–12, 2013. doi:10.1016/j.nbd.2011.12.057. 22266017 PMC3337963

[12] Guardia-Laguarta C, Area-Gomez E, Schon EA, Przedborski S. A new role for α-synuclein in Parkinson’s disease: Alteration of ER–mitochondrial communication. Mov Disord 30: 1026–1033, 2015. doi:10.1002/mds.26239. 25952565

[13] Benzi G, Pastoris O, Marzatico F, Villa RF, Dagani F, Curti D. The mitochondrial electron transfer alteration as a factor involved in the brain aging. Neurobiol Aging 13: 361–368, 1992. doi:10.1016/0197-4580(92)90109-B. 1320745

[14] Monzio Compagnoni G, Di Fonzo A, Corti S, Comi GP, Bresolin N, Masliah E. The role of mitochondria in neurodegenerative diseases: the lesson from Alzheimer’s disease and Parkinson’s disease. Mol Neurobiol 57: 2959–2980, 2020. doi:10.1007/s12035-020-01926-1. 32445085 PMC9047992

[15] Damiano M, Diguet E, Malgorn C, D'Aurelio M, Galvan L, Petit F, Benhaim L, Guillermier M, Houitte D, Dufour N, Hantraye P, Canals JM, Alberch J, Delzescaux T, Déglon N, Beal MF, Brouillet E. A role of mitochondrial complex II defects in genetic models of Huntington’s disease expressing *N*-terminal fragments of mutant huntingtin. Human Molecular Genetics 22: 3869–3882, 2013. doi:10.1093/hmg/ddt242. 23720495 PMC3766181

[16] Navarro A, Boveris A. Brain mitochondrial dysfunction in aging, neurodegeneration, and Parkinson’s disease. Front Aging Neurosci 2: 34, 2010. doi:10.3389/fnagi.2010.00034. 20890446 PMC2947925

[17] Lin MT, Beal MF. Mitochondrial dysfunction and oxidative stress in neurodegenerative diseases. Nature 443: 787–795, 2006. doi:10.1038/nature05292. 17051205

[18] Burtscher J, Millet GP, Place N, Kayser B, Zanou N. The muscle-brain axis and neurodegenerative diseases: the key role of mitochondria in exercise-induced neuroprotection. International Journal of Molecular Sciences 22: 6479, 2021. doi:10.3390/ijms22126479. 34204228 PMC8235687

[19] Sun N, Yun J, Liu J, Malide D, Liu C, Rovira II, Holmström KM, Fergusson MM, Yoo YH, Combs CA, Finkel T. Measuring in vivo mitophagy. Mol Cell 60: 685–696, 2015. doi:10.1016/j.molcel.2015.10.009. 26549682 PMC4656081

[20] Nixon RA. The role of autophagy in neurodegenerative disease. Nat Med 19: 983–997, 2013. doi:10.1038/nm.3232. 23921753

[21] Ryan BJ, Hoek S, Fon EA, Wade-Martins R. Mitochondrial dysfunction and mitophagy in Parkinson’s: from familial to sporadic disease. Trends Biochem Sci 40: 200–210, 2015. doi:10.1016/j.tibs.2015.02.003. 25757399

[22] Sheng B, Wang X, Su B, Lee H, Casadesus G, Perry G, Zhu X. Impaired mitochondrial biogenesis contributes to mitochondrial dysfunction in Alzheimer’s disease. J Neurochem 120: 419–429, 2012. doi:10.1111/j.1471-4159.2011.07581.x. 22077634 PMC3253532

[23] Ryan SD, Dolatabadi N, Chan SF, Zhang X, Akhtar MW, Parker J, Soldner F, Sunico CR, Nagar S, Talantova M, Lee B, Lopez K, Nutter A, Shan B, Molokanova E, Zhang Y, Han X, Nakamura T, Masliah E, Yates JR, Nakanishi N, Andreyev AY, Okamoto S, Jaenisch R, Ambasudhan R, Lipton SA. Isogenic human iPSC Parkinson’s model shows nitrosative stress-induced dysfunction in MEF2-PGC1α transcription. Cell 155: 1351–1364, 2013 [Erratum in *Cell* 155: 1652–1653, 2013]. doi:10.1016/j.cell.2013.11.009. 24290359 PMC4028128

[24] Batista AF, Rody T, Forny-Germano L, Cerdeiro S, Bellio M, Ferreira ST, Munoz DP, De Felice FG. Interleukin-1β mediates alterations in mitochondrial fusion/fission proteins and memory impairment induced by amyloid-β oligomers. J Neuroinflammation 18: 54, 2021. doi:10.1186/s12974-021-02099-x. 33612100 PMC7897381

[25] Gao J, Wang L, Liu J, Xie F, Su B, Wang X. Abnormalities of mitochondrial dynamics in neurodegenerative diseases. Antioxidants (Basel) 6: 25, 2017. doi:10.3390/antiox6020025. 28379197 PMC5488005

[26] Carelli V, Musumeci O, Caporali L, Zanna C, La Morgia C, Del Dotto V, Porcelli AM, Rugolo M, Valentino ML, Iommarini L, Maresca A, Barboni P, Carbonelli M, Trombetta C, Valente EM, Patergnani S, Giorgi C, Pinton P, Rizzo G, Tonon C, Lodi R, Avoni P, Liguori R, Baruzzi A, Toscano A, Zeviani M. Syndromic parkinsonism and dementia associated with OPA1 missense mutations. Ann Neurol 78: 21–38, 2015. doi:10.1002/ana.24410. 25820230 PMC5008165

[27] Kann O, Kovács R. Mitochondria and neuronal activity. Am J Physiol Cell Physiol 292: C641–C657, 2007. doi:10.1152/ajpcell.00222.2006. 17092996

[28] Magistretti PJ, Allaman I. A Cellular Perspective on Brain Energy Metabolism and Functional Imaging. Neuron 86: 883–901, 2015. doi:10.1016/j.neuron.2015.03.035. 25996133

[29] Schönfeld P, Reiser G. Why does brain metabolism not favor burning of fatty acids to provide energy? - reflections on disadvantages of the use of free fatty acids as fuel for brain. J Cereb Blood Flow Metab 33: 1493–1499, 2013. doi:10.1038/jcbfm.2013.128. 23921897 PMC3790936

[30] Zheng X, Boyer L, Jin M, Mertens J, Kim Y, Ma L, Ma L, Hamm M, Gage FH, Hunter T. Metabolic reprogramming during neuronal differentiation from aerobic glycolysis to neuronal oxidative phosphorylation. eLife 5: e13374, 2016. doi:10.7554/eLife.13374. 27282387 PMC4963198

[31] Díaz-García CM, Mongeon R, Lahmann C, Koveal D, Zucker H, Yellen G. Neuronal stimulation triggers neuronal glycolysis and not lactate uptake. Cell Metab 26: 361–374.e4, 2017. doi:10.1016/j.cmet.2017.06.021. 28768175 PMC5559896

[32] Groten CJ, MacVicar BA. Mitochondrial Ca^2+^ uptake by the MCU facilitates pyramidal neuron excitability and metabolism during action potential firing. Commun Biol 5: 900, 2022. doi:10.1038/s42003-022-03848-1. 36056095 PMC9440007

[33] Pernas L, Scorrano L. Mito-morphosis: mitochondrial fusion, fission, and cristae remodeling as key mediators of cellular function. Annu Rev Physiol 78: 505–531, 2016. doi:10.1146/annurev-physiol-021115-105011. 26667075

[34] Bélanger M, Allaman I, Magistretti PJ. Brain energy metabolism: focus on astrocyte-neuron metabolic cooperation. Cell Metab 14: 724–738, 2011. doi:10.1016/j.cmet.2011.08.016. 22152301

[35] Bouzier-Sore A-K, Pellerin L. Unraveling the complex metabolic nature of astrocytes [Online]. Front Cell Neurosci 7: 179, 2013. doi:10.3389/fncel.2013.00179. 24130515 PMC3795301

[36] Bolaños JP. Bioenergetics and redox adaptations of astrocytes to neuronal activity. J Neurochem 139, *Suppl* 2: 115–125, 2016. doi:10.1111/jnc.13486. 26968531 PMC5018236

[37] Zhang J, Malik A, Choi HB, Ko RWY, Dissing-Olesen L, MacVicar BA. Microglial CR3 activation triggers long-term synaptic depression in the hippocampus via NADPH oxidase. Neuron 82: 195–207, 2014. doi:10.1016/j.neuron.2014.01.043. 24631344

[38] Aldana BI. Microglia-specific metabolic changes in neurodegeneration. J Mol Biol 431: 1830–1842, 2019. doi:10.1016/j.jmb.2019.03.006. 30878483

[39] Miller BF, Hamilton KL. A perspective on the determination of mitochondrial biogenesis. Am J Physiol Endocrinol Physiol 302: E496–E499, 2012. doi:10.1152/ajpendo.00578.2011. 22205627 PMC3311289

[40] Miller BF, Reid JJ, Price JC, Lin H-JL, Atherton PJ, Smith K. CORP: the use of deuterated water for the measurement of protein synthesis. J Appl Physiol (1985) 128: 1163–1176, 2020. doi:10.1152/japplphysiol.00855.2019. 32213116

[41] Reid JJ, Linden MA, Peelor FF, Miller RA, Hamilton KL, Miller BF. Brain protein synthesis rates in the UM-HET3 mouse following treatment with rapamycin or rapamycin with metformin. J Gerontol A Biol Sci Med Sci 75: 40–49, 2020. doi:10.1093/gerona/glz069. 30864661 PMC7175973

[42] Price JC, Guan S, Burlingame A, Prusiner SB, Ghaemmaghami S. Analysis of proteome dynamics in the mouse brain. Proc Natl Acad Sci USA 107: 14508–14513, 2010 [Erratum in *Proc Natl Acad Sci USA* 111: 3645, 2014]. doi:10.1073/pnas.1006551107. 20699386 PMC2922600

[43] Kobak KA, Lawrence MM, Pharaoh G, Borowik AK, Peelor FF, Shipman PD, Griffin TM, Van Remmen H, Miller BF. Determining the contributions of protein synthesis and breakdown to muscle atrophy requires non-steady-state equations. J Cachexia Sarcopenia Muscle 12: 1764–1775, 2021. doi:10.1002/jcsm.12772. 34418329 PMC8718081

[44] Bayraktar EC, Baudrier L, Özerdem C, Lewis CA, Chan SH, Kunchok T, Abu-Remaileh M, Cangelosi AL, Sabatini DM, Birsoy K, Chen WW. MITO-Tag Mice enable rapid isolation and multimodal profiling of mitochondria from specific cell types in vivo. Proc Natl Acad Sci USA 116: 303–312, 2019. doi:10.1073/pnas.1816656115. 30541894 PMC6320505

[45] Fecher C, Trovò L, Müller SA, Snaidero N, Wettmarshausen J, Heink S, Ortiz O, Wagner I, Kühn R, Hartmann J, Karl RM, Konnerth A, Korn T, Wurst W, Merkler D, Lichtenthaler SF, Perocchi F, Misgeld T. Cell-type-specific profiling of brain mitochondria reveals functional and molecular diversity. Nat Neurosci 22: 1731–1742, 2019. doi:10.1038/s41593-019-0479-z. 31501572

[46] Bulovaite E, Qiu Z, Kratschke M, Zgraj A, Fricker DG, Tuck EJ, Gokhale R, Koniaris B, Jami SA, Merino-Serrais P, Husi E, Mendive-Tapia L, Vendrell M, O’Dell TJ, DeFelipe J, Komiyama NH, Holtmaat A, Fransén E, Grant SGN. A brain atlas of synapse protein lifetime across the mouse lifespan. Neuron 110: 4057–4073.e8, 2022. doi:10.1016/j.neuron.2022.09.009. 36202095 PMC9789179

[47] López-Doménech G, Covill-Cooke C, Ivankovic D, Halff EF, Sheehan DF, Norkett R, Birsa N, Kittler JT. Miro proteins coordinate microtubule- and actin-dependent mitochondrial transport and distribution. EMBO J 37: 321–336, 2018. doi:10.15252/embj.201696380. 29311115 PMC5793800

[48] Russo GJ, Louie K, Wellington A, Macleod GT, Hu F, Panchumarthi S, Zinsmaier KE. Drosophila Miro is required for both anterograde and retrograde axonal mitochondrial transport. J Neurosci 29: 5443–5455, 2009. doi:10.1523/JNEUROSCI.5417-08.2009. 19403812 PMC2693725

[49] Stowers RS, Megeath LJ, Górska-Andrzejak J, Meinertzhagen IA, Schwarz TL. Axonal transport of mitochondria to synapses depends on milton, a novel Drosophila protein. Neuron 36: 1063–1077, 2002. doi:10.1016/s0896-6273(02)01094-2. 12495622

[50] Lee Y, Jeong S-Y, Karbowski M, Smith CL, Youle RJ. Roles of the mammalian mitochondrial fission and fusion mediators Fis1, Drp1, and Opa1 in apoptosis. Mol Biol Cell 15: 5001–5011, 2004. doi:10.1091/mbc.e04-04-0294. 15356267 PMC524759

[51] Chen H, Detmer SA, Ewald AJ, Griffin EE, Fraser SE, Chan DC. Mitofusins Mfn1 and Mfn2 coordinately regulate mitochondrial fusion and are essential for embryonic development. J Cell Biol 160: 189–200, 2003. doi:10.1083/jcb.200211046. 12527753 PMC2172648

[52] Pitts KR, Yoon Y, Krueger EW, McNiven MA. The dynamin-like protein DLP1 is essential for normal distribution and morphology of the endoplasmic reticulum and mitochondria in mammalian cells. Mol Biol Cell 10: 4403–4417, 1999. doi:10.1091/mbc.10.12.4403. 10588666 PMC25766

[53] Losón OC, Song Z, Chen H, Chan DC. Fis1, Mff, MiD49, and MiD51 mediate Drp1 recruitment in mitochondrial fission. Mol Biol Cell 24: 659–667, 2013. doi:10.1091/mbc.E12-10-0721. 23283981 PMC3583668

[54] Gandre-Babbe S, van der Bliek AM. The novel tail-anchored membrane protein Mff controls mitochondrial and peroxisomal fission in mammalian cells. Mol Biol Cell 19: 2402–2412, 2008. doi:10.1091/mbc.E07-12-1287. 18353969 PMC2397315

[55] Palmer CS, Elgass KD, Parton RG, Osellame LD, Stojanovski D, Ryan MT. Adaptor proteins MiD49 and MiD51 can act independently of Mff and Fis1 in Drp1 recruitment and are specific for mitochondrial fission. J Biol Chem 288: 27584–27593, 2013. doi:10.1074/jbc.M113.479873. 23921378 PMC3779755

[56] Buck MD, O'Sullivan D, Klein Geltink RI, Curtis JD, Chang C-H, Sanin DE, Qiu J, Kretz O, Braas D, van der Windt GJW, Chen Q, Huang SC-C, O'Neill CM, Edelson BT, Pearce EJ, Sesaki H, Huber TB, Rambold AS, Pearce EL. Mitochondrial dynamics controls T cell fate through metabolic programming. Cell 166: 63–76, 2016. doi:10.1016/j.cell.2016.05.035. 27293185 PMC4974356

[57] D'Amico D, Mottis A, Potenza F, Sorrentino V, Li H, Romani M, Lemos V, Schoonjans K, Zamboni N, Knott G, Schneider BL, Auwerx J. The RNA-binding protein PUM2 impairs mitochondrial dynamics and mitophagy during aging. Mol Cell 73: 775–787.e10, 2019. doi:10.1016/j.molcel.2018.11.034. 30642763 PMC6396316

[58] Popov V, Medvedev NI, Davies HA, Stewart MG. Mitochondria form a filamentous reticular network in hippocampal dendrites but are present as discrete bodies in axons: a three-dimensional ultrastructural study. J Comp Neurol 492: 50–65, 2005. doi:10.1002/cne.20682. 16175555

[59] Zhang L, Trushin S, Christensen TA, Bachmeier BV, Gateno B, Schroeder A, Yao J, Itoh K, Sesaki H, Poon WW, Gylys KH, Patterson ER, Parisi JE, Diaz Brinton R, Salisbury JL, Trushina E. Altered brain energetics induces mitochondrial fission arrest in Alzheimer’s Disease. Sci Rep 6: 18725, 2016. doi:10.1038/srep18725. 26729583 PMC4700525

[60] Mitra K, Lippincott-Schwartz J. Analysis of mitochondrial dynamics and functions using imaging approaches. Curr Protoc Cell Biol 46: 4.25.1– 4.25.21, 2010. doi:10.1002/0471143030.cb0425s46. 20235105 PMC3007120

[61] Karbowski M, Arnoult D, Chen H, Chan DC, Smith CL, Youle RJ. Quantitation of mitochondrial dynamics by photolabeling of individual organelles shows that mitochondrial fusion is blocked during the Bax activation phase of apoptosis. J Cell Biol 164: 493–499, 2004. doi:10.1083/jcb.200309082. 14769861 PMC2172000

[62] Twig G, Graf SA, Wikstrom JD, Mohamed H, Haigh SE, Elorza A, Deutsch M, Zurgil N, Reynolds N, Shirihai OS. Tagging and tracking individual networks within a complex mitochondrial web with photoactivatable GFP. Am J Physiol Cell Physiol 291: C176–C184, 2006. doi:10.1152/ajpcell.00348.2005. 16481372

[63] Beattie DS, Basford RE, Koritz SB. The turnover of the protein components of mitochondria from rat liver, kidney, and brain. J Biol Chem 242: 4584–4586, 1967. doi:10.1016/S0021-9258(18)99496-2. 6061404

[64] Kim T-Y, Wang D, Kim AK, Lau E, Lin AJ, Liem DA, Zhang J, Zong NC, Lam MPY, Ping P. Metabolic labeling reveals proteome dynamics of mouse mitochondria. Mol Cell Proteomics 11: 1586–1594, 2012. doi:10.1074/mcp.M112.021162. 22915825 PMC3518123

[65] Fornasiero EF, Mandad S, Wildhagen H, Alevra M, Rammner B, Keihani S, Opazo F, Urban I, Ischebeck T, Sakib MS, Fard MK, Kirli K, Centeno TP, Vidal RO, Rahman R-U, Benito E, Fischer A, Dennerlein S, Rehling P, Feussner I, Bonn S, Simons M, Urlaub H, Rizzoli SO. Precisely measured protein lifetimes in the mouse brain reveal differences across tissues and subcellular fractions. Nat Commun 9: 4230, 2018. doi:10.1038/s41467-018-06519-0. 30315172 PMC6185916

[66] Ashrafi G, Schlehe JS, LaVoie MJ, Schwarz TL. Mitophagy of damaged mitochondria occurs locally in distal neuronal axons and requires PINK1 and Parkin. J Cell Biol 206: 655–670, 2014. doi:10.1083/jcb.201401070. 25154397 PMC4151150

[67] Bomba-Warczak E, Edassery SL, Hark TJ, Savas JN. Long-lived mitochondrial cristae proteins in mouse heart and brain. J Cell Biol 220: e202005193, 2021. doi:10.1083/jcb.202005193. 34259807 PMC8282663

[68] Toyama BH, Savas JN, Park SK, Harris MS, Ingolia NT, Yates JR, Hetzer MW. Identification of long-lived proteins reveals exceptional stability of essential cellular structures. Cell 154: 971–982, 2013. doi:10.1016/j.cell.2013.07.037. 23993091 PMC3788602

[69] Dörrbaum AR, Kochen L, Langer JD, Schuman EM. Local and global influences on protein turnover in neurons and glia. eLife 7: e34202, 2018. doi:10.7554/eLife.34202. 29914620 PMC6008053

[70] Jackson JG, O'Donnell JC, Takano H, Coulter DA, Robinson MB. Neuronal activity and glutamate uptake decrease mitochondrial mobility in astrocytes and position mitochondria near glutamate transporters. J Neurosci 34: 1613–1624, 2014. doi:10.1523/JNEUROSCI.3510-13.2014. 24478345 PMC3905137

[71] Zehnder T, Petrelli F, Romanos J, De Oliveira Figueiredo EC, Lewis TL, Déglon N, Polleux F, Santello M, Bezzi P. Mitochondrial biogenesis in developing astrocytes regulates astrocyte maturation and synapse formation. Cell Rep 35: 108952, 2021. doi:10.1016/j.celrep.2021.108952. 33852851

[72] Chang DTW, Reynolds IJ. Differences in mitochondrial movement and morphology in young and mature primary cortical neurons in culture. Neuroscience 141: 727–736, 2006. doi:10.1016/j.neuroscience.2006.01.034. 16797853

[73] Lewis TL, Kwon S-K, Lee A, Shaw R, Polleux F. MFF-dependent mitochondrial fission regulates presynaptic release and axon branching by limiting axonal mitochondria size. Nat Commun 9: 5008, 2018. doi:10.1038/s41467-018-07416-2. 30479337 PMC6258764

[74] Smit-Rigter L, Rajendran R, Silva CAP, Spierenburg L, Groeneweg F, Ruimschotel EM, van Versendaal D, van der Togt C, Eysel UT, Heimel JA, Lohmann C, Levelt CN. Mitochondrial dynamics in visual cortex are limited in vivo and not affected by axonal structural plasticity. Curr Biol 26: 2609–2616, 2016. doi:10.1016/j.cub.2016.07.033. 27641766

[75] Lewis TL, Turi GF, Kwon S-K, Losonczy A, Polleux F. Progressive decrease of mitochondrial motility during maturation of cortical axons in vitro and in vivo. Curr Biol 26: 2602–2608, 2016. doi:10.1016/j.cub.2016.07.064. 27641765 PMC5235338

[76] Wang X, Su B, Lee H, Li X, Perry G, Smith MA, Zhu X. Impaired balance of mitochondrial fission and fusion in Alzheimer’s Disease. J Neurosci 29: 9090–9103, 2009. doi:10.1523/JNEUROSCI.1357-09.2009. 19605646 PMC2735241

[77] Santos RX, Correia SC, Wang X, Perry G, Smith MA, Moreira PI, Zhu X. A synergistic dysfunction of mitochondrial fission/fusion dynamics and mitophagy in Alzheimer’s disease. JAD 20: S401–S412, 2010. doi:10.3233/JAD-2010-100666. 20463393 PMC2923835

[78] Cheng Q, Chen J, Guo H, Lu J-L, Zhou J, Guo X-Y, Shi Y, Zhang Y, Yu S, Zhang Q, Ding F. Pyrroloquinoline quinone promotes mitochondrial biogenesis in rotenone-induced Parkinson’s disease model via AMPK activation. Acta Pharmacol Sin 42: 665–678, 2021. doi:10.1038/s41401-020-0487-2. 32860006 PMC8115282

[79] Chen H, McCaffery JM, Chan DC. Mitochondrial fusion protects against neurodegeneration in the cerebellum. Cell 130: 548–562, 2007. doi:10.1016/j.cell.2007.06.026. 17693261

[80] Olichon A, Baricault L, Gas N, Guillou E, Valette A, Belenguer P, Lenaers G. Loss of OPA1 perturbates the mitochondrial inner membrane structure and integrity, leading to cytochrome c release and apoptosis. J Biol Chem 278: 7743–7746, 2003. doi:10.1074/jbc.C200677200. 12509422

[81] Lee A, Kondapalli C, Virga DM, Lewis TL, Koo SY, Ashok A, Mairet-Coello G, Herzig S, Foretz M, Viollet B, Shaw R, Sproul A, Polleux F. Aβ42 oligomers trigger synaptic loss through CAMKK2-AMPK-dependent effectors coordinating mitochondrial fission and mitophagy. Nat Commun 13: 4444, 2022. doi:10.1038/s41467-022-32130-5. 35915085 PMC9343354

[82] Calkins MJ, Manczak M, Mao P, Shirendeb U, Reddy PH. Impaired mitochondrial biogenesis, defective axonal transport of mitochondria, abnormal mitochondrial dynamics and synaptic degeneration in a mouse model of Alzheimer’s disease. Hum Mol Genet 20: 4515–4529, 2011. doi:10.1093/hmg/ddr381. 21873260 PMC3209824

[83] Neufer PD, Bamman MM, Muoio DM, Bouchard C, Cooper DM, Goodpaster BH, Booth FW, Kohrt WM, Gerszten RE, Mattson MP, Hepple RT, Kraus WE, Reid MB, Bodine SC, Jakicic JM, Fleg JL, Williams JP, Joseph L, Evans M, Maruvada P, Rodgers M, Roary M, Boyce AT, Drugan JK, Koenig JI, Ingraham RH, Krotoski D, Garcia-Cazarin M, McGowan JA, Laughlin MR. Understanding the cellular and molecular mechanisms of physical activity-induced health benefits. Cell Metab 22: 4–11, 2015. doi:10.1016/j.cmet.2015.05.011. 26073496

[84] Radak Z, Hart N, Sarga L, Koltai E, Atalay M, Ohno H, Boldogh I. Exercise plays a preventive role against Alzheimer’s disease. J Alzheimers Dis 20: 777–783, 2010. doi:10.3233/JAD-2010-091531. 20182027

[85] Colcombe SJ, Erickson KI, Scalf PE, Kim JS, Prakash R, McAuley E, Elavsky S, Marquez DX, Hu L, Kramer AF. Aerobic exercise training increases brain volume in aging humans. J Gerontol A Biol Sci Med Sci 61: 1166–1170, 2006. doi:10.1093/gerona/61.11.1166. 17167157

[86] Rhodes JS, van Praag H, Jeffrey S, Girard I, Mitchell GS, Garland T, Jr, Gage FH. Exercise increases hippocampal neurogenesis to high levels but does not improve spatial learning in mice bred for increased voluntary wheel running. Behav Neurosci 117: 1006–1016, 2003 [Erratum in *Behav Neurosci* 118: 305, 2004]. doi:10.1037/0735-7044.117.5.1006. 14570550

[87] Barnes DE, Yaffe K, Satariano WA, Tager IB. A longitudinal study of cardiorespiratory fitness and cognitive function in healthy older adults. J Am Geriatr Soc 51: 459–465, 2003. doi:10.1046/j.1532-5415.2003.51153.x. 12657064

[88] Valenzuela PL, Morales JS, Castillo-García A, Mayordomo-Cava J, García-Hermoso A, Izquierdo M, Serra-Rexach JA, Lucia A. Effects of exercise interventions on the functional status of acutely hospitalised older adults: a systematic review and meta-analysis. Ageing Res Rev 61: 101076, 2020. doi:10.1016/j.arr.2020.101076. 32330558

[89] Aberg MAI, Pedersen NL, Torén K, Svartengren M, Bäckstrand B, Johnsson T, Cooper-Kuhn CM, Aberg ND, Nilsson M, Kuhn HG. Cardiovascular fitness is associated with cognition in young adulthood. Proc Natl Acad Sci USA 106: 20906–20911, 2009. doi:10.1073/pnas.0905307106. 19948959 PMC2785721

[90] Erickson KI, Voss MW, Prakash RS, Basak C, Szabo A, Chaddock L, Kim JS, Heo S, Alves H, White SM, Wojcicki TR, Mailey E, Vieira VJ, Martin SA, Pence BD, Woods JA, McAuley E, Kramer AF. Exercise training increases size of hippocampus and improves memory. Proc Natl Acad Sci USA 108: 3017–3022, 2011. doi:10.1073/pnas.1015950108. 21282661 PMC3041121

[91] Dietrich MO, Andrews ZB, Horvath TL. Exercise-induced synaptogenesis in the hippocampus is dependent on UCP2-regulated mitochondrial adaptation. J Neurosci 28: 10766–10771, 2008. doi:10.1523/JNEUROSCI.2744-08.2008. 18923051 PMC3865437

[92] van Praag H, Christie BR, Sejnowski TJ, Gage FH. Running enhances neurogenesis, learning, and long-term potentiation in mice. Proc Natl Acad Sci USA 96: 13427–13431, 1999. doi:10.1073/pnas.96.23.13427. 10557337 PMC23964

[93] Clark PJ, Kohman RA, Miller DS, Bhattacharya TK, Brzezinska WJ, Rhodes JS. Genetic influences on exercise-induced adult hippocampal neurogenesis across 12 divergent mouse strains. Genes Brain Behav 10: 345–353, 2011. doi:10.1111/j.1601-183X.2010.00674.x. 21223504 PMC3139814

[94] Navarro A, Boveris A. Rat brain and liver mitochondria develop oxidative stress and lose enzymatic activities on aging. Am J Physiol Regul Integr Comp Physiol 287: R1244–R1249, 2004. doi:10.1152/ajpregu.00226.2004. 15271654

[95] Kirchner L, Chen W-Q, Afjehi-Sadat L, Viidik A, Skalicky M, Höger H, Lubec G. Hippocampal metabolic proteins are modulated in voluntary and treadmill exercise rats. Exp Neurol 212: 145–151, 2008. doi:10.1016/j.expneurol.2008.03.014. 18455160

[96] Bernardo T. C, Marques-Aleixo I, Beleza J, Oliveira P. J, Ascensão A, Magalhães J. Physical exercise and brain mitochondrial fitness: the possible role against Alzheimer’s disease. Brain Pathol 26: 648–663, 2016. doi:10.1111/bpa.12403. 27328058 PMC8029062

[97] Geda YE, Roberts RO, Knopman DS, Christianson TJH, Pankratz VS, Ivnik RJ, Boeve BF, Tangalos EG, Petersen RC, Rocca WA. Physical exercise, aging, and mild cognitive impairment: a population-based study. Arch Neurol 67: 80–86, 2010. doi:10.1001/archneurol.2009.297. 20065133 PMC2919839

[98] Xu Q, Park Y, Huang X, Hollenbeck A, Blair A, Schatzkin A, Chen H. Physical activities and future risk of Parkinson disease. Neurology 75: 341–348, 2010. doi:10.1212/WNL.0b013e3181ea1597. 20660864 PMC2918886

[99] Liang KY, Mintun MA, Fagan AM, Goate AM, Bugg JM, Holtzman DM, Morris JC, Head D. Exercise and Alzheimer’s disease biomarkers in cognitively normal older adults. Ann Neurol 68: 311–318, 2010. doi:10.1002/ana.22096. 20818789 PMC2936720

[100] Adlard PA, Perreau VM, Pop V, Cotman CW. Voluntary exercise decreases amyloid load in a transgenic model of Alzheimer’s disease. J Neurosci 25: 4217–4221, 2005. doi:10.1523/JNEUROSCI.0496-05.2005. 15858047 PMC6725122

[101] Bo H, Kang W, Jiang N, Wang X, Zhang Y, Ji LL. Exercise-induced neuroprotection of hippocampus in APP/PS1 transgenic mice via upregulation of mitochondrial 8-oxoguanine DNA glycosylase. Oxid Med Cell Longev 2014: 834502–834514, 2014. doi:10.1155/2014/834502. 25538817 PMC4236906

[102] Lau Y-S, Patki G, Das-Panja K, Le W-D, Ahmad SO. Neuroprotective effects and mechanisms of exercise in a chronic mouse model of Parkinson’s disease with moderate neurodegeneration. Eur J Neurosci 33: 1264–1274, 2011. doi:10.1111/j.1460-9568.2011.07626.x. 21375602 PMC3079264

[103] Liu Y, Chu JMT, Ran Y, Zhang Y, Chang RCC, Wong GTC. Prehabilitative resistance exercise reduces neuroinflammation and improves mitochondrial health in aged mice with perioperative neurocognitive disorders. J Neuroinflammation 19: 150, 2022. doi:10.1186/s12974-022-02483-1. 35705955 PMC9199135

[104] Liu Y, Chu JMT, Yan T, Zhang Y, Chen Y, Chang RCC, Wong GTC. Short-term resistance exercise inhibits neuroinflammation and attenuates neuropathological changes in 3xTg Alzheimer’s disease mice. J Neuroinflammation 17: 4, 2020. doi:10.1186/s12974-019-1653-7. 31900170 PMC6942350

[105] Dungan CM, Valentino T, Vechetti IJ, Zdunek CJ, Murphy MP, Lin A-L, McCarthy JJ, Peterson CA. Exercise-mediated alteration of hippocampal Dicer mRNA and miRNAs is associated with lower BACE1 gene expression and Aβ1-42 in female 3xTg-AD mice. J Neurophysiol 124: 1571–1577, 2020. doi:10.1152/jn.00503.2020. 33052800 PMC7814900

[106] Steiner JL, Murphy EA, McClellan JL, Carmichael MD, Davis JM. Exercise training increases mitochondrial biogenesis in the brain. J Appl Physiol (1985) 111: 1066–1071, 2011. doi:10.1152/japplphysiol.00343.2011. 21817111

[107] Zhang Q, Wu Y, Zhang P, Sha H, Jia J, Hu Y, Zhu J. Exercise induces mitochondrial biogenesis after brain ischemia in rats. Neuroscience 205: 10–17, 2012. doi:10.1016/j.neuroscience.2011.12.053. 22266265

[108] E L, Burns JM, Swerdlow RH. Effect of high-intensity exercise on aged mouse brain mitochondria, neurogenesis, and inflammation. Neurobiol Aging 35: 2574–2583, 2014. doi:10.1016/j.neurobiolaging.2014.05.033. 25002036 PMC4171347

[109] Park J, Kim J, Mikami T. Exercise-induced lactate release mediates mitochondrial biogenesis in the hippocampus of mice via monocarboxylate transporters. Front Physiol 12: 736905, 2021. doi:10.3389/fphys.2021.736905. 34603087 PMC8481603

[110] Chuang C-S, Chang J-C, Cheng F-C, Liu K-H, Su H-L, Liu C-S. Modulation of mitochondrial dynamics by treadmill training to improve gait and mitochondrial deficiency in a rat model of Parkinson’s disease. Life Sci 191: 236–244, 2017. doi:10.1016/j.lfs.2017.10.003. 28986095

[111] Koo J-H, Kang E-B. Effects of treadmill exercise on the regulatory mechanisms of mitochondrial dynamics and oxidative stress in the brains of high-fat diet fed rats. J Exerc Nutrition Biochem 23: 28–35, 2019. doi:10.20463/jenb.2019.0005. 31010272 PMC6477818

[112] Yan Q-W, Zhao N, Xia J, Li B-X, Yin L-Y. Effects of treadmill exercise on mitochondrial fusion and fission in the hippocampus of APP/PS1 mice. Neurosci Lett 701: 84–91, 2019. doi:10.1016/j.neulet.2019.02.030. 30796962

[113] Burman JL, Pickles S, Wang C, Sekine S, Vargas JNS, Zhang Z, Youle AM, Nezich CL, Wu X, Hammer JA, Youle RJ. Mitochondrial fission facilitates the selective mitophagy of protein aggregates. J Cell Biol 216: 3231–3247, 2017. doi:10.1083/jcb.201612106. 28893839 PMC5626535

[114] Fang EF, Hou Y, Palikaras K, Adriaanse BA, Kerr JS, Yang B, Lautrup S, Hasan-Olive MM, Caponio D, Dan X, Rocktäschel P, Croteau DL, Akbari M, Greig NH, Fladby T, Nilsen H, Cader MZ, Mattson MP, Tavernarakis N, Bohr VA. Mitophagy inhibits amyloid-β and tau pathology and reverses cognitive deficits in models of Alzheimer’s disease. Nat Neurosci 22: 401–412, 2019. doi:10.1038/s41593-018-0332-9. 30742114 PMC6693625

